# Can left internal mammary artery side branches affect blood flow rate?

**Published:** 2009-04

**Authors:** S Biceroglu, M Karaca, M Ildizli Demirbas, H Yilmaz, A Yildiz

**Affiliations:** Atakalp Heart Center, Izmir, Turkey; Atakalp Heart Center, Izmir, Turkey; Atakalp Heart Center, Izmir, Turkey; Atakalp Heart Center, Izmir, Turkey; Gazi Hospital, Department of Cardiology, Izmir, Turkey

## Abstract

**Summary:**

The left internal mammary artery (LIMA) is the most commonly used arterial graft for coronary artery bypass graft (CABG) surgery, and occluding the LIMA side branches during surgery is important to avoid myocardial ischaemia afterwards. In this study we investigated the incidence of patent LIMA graft side branches in our coronary angiography series, and compared LIMA flow rate changes by means of the thrombolysis in myocardial infarction (TIMI) frame count in patients with and without LIMA graft side branches.

Patients with a history of CABG surgery and who were scheduled for coronary angiography in our centre between 1 January and 15 December 2006 were enrolled in the study. We compared LIMA graft TIMI frame counts between patients with and without side branches. The incidence of LIMA graft side branches in our study was 18% (seven patients). Mean TIMI frame count was 27.28 ± 3.4 in patients with LIMA graft side branches and 15.67 ± 2.3 in patients without. There was a significant difference between the two groups (*p* < 0.0001). Patients with LIMA graft side branches were more likely to have anterior ischaemia, determined by myocardial perfusion scintigraphy.

We suggest that TIMI frame count may be helpful in evaluating the effect of side branches on LIMA graft flow rate. The increased TIMI frame count of a LIMA graft with side branch is associated with insufficient LIMA flow.

## Summary

Left internal mammary artery (LIMA) side branches to the chest wall (pectoralis branch) are common, occurring in 9–25% of patients who have undergone CABG surgery.^1^,^2^ The idea that a large side branch from a LIMA bypass graft can cause ischaemia by ‘stealing’ blood flow and directing it to the left anterior descending coronary artery is the subject of continued controversy. Large side branches are capable of substantial run off, which can compromise flow to the distal internal mammary artery (IMA). This may be due to the relatively lower peripheral resistance compared with coronary resistance. Angina occasionally occurs after coronary artery bypass grafting (CABG) and may be the result of coronary diversion when large side-branch thoracic arteries originate from the internal thoracic artery.^3^

The LIMA side branch may be occluded during surgery to help prevent myocardial ischaemia.^2^ This can be done using several techniques, including transcatheter embolisation and surgical ligature. These techniques have been reported to provide symptomatic relief.^4^-^6^ Coil occlusion is a recently described method that can be used to occlude the side branch.^7^,^8^

Clinical success depends on the demonstration of myocardial ischaemia before occlusion of the LIMA side branch. Myocardial ischaemia is most often identified using myocardial perfusion scintigraphy, but Doppler evaluation is a good alternative.^9^ Many reports measure angiographic diameter of the LIMA and/or left anterior descending artery before and after side-branch occlusion as a surrogate measurement of increased blood flow.^10^

In this study, we investigated the incidence of LIMA side branches in our coronary angiography series. LIMA flow rate changes were evaluated using the thrombolysis in myocardial infarction (TIMI) frame count to assess coronary perfusion.

## Methods

Patients with a history of CABG surgery who were scheduled for coronary angiography at our centre between January and December 2006 were retrospectively enrolled in the study. All patients had chest pain and myocardial ischaemia, demonstrated by electrocardiogram (ECG) changes, myocardial perfusion scintigraphy or treadmill exercise testing. Patients with saphenous grafts for left anterior descending artery (LAD) revascularisation, insufficient angiographic image quality or occluded LIMA grafts were excluded from this study. Clinical data were obtained from the hospital’s archives with the permission of the local ethics comittee.

Two cardiologists evaluated all coronary angiograms. The incidence of LIMA side branches, their origin, destination and properties were noted. The LIMA was divided visually into three equal parts. The diameter of each LIMA side branch was measured. All measurements were made from the proximal one-third of the LIMA and the first 1 cm of the side branch.

Quantitative measurements of coronary blood flow were made by measuring the elapsed time from the appearance of a contrast agent until it reached the distal end of the LIMA. The measured ciné frame count was considered the TIMI frame count. The final count was subtracted from the initial count, and the exact TIMI frame count was calculated. We additionally compared LIMA frame counts between patients with and without side branches.

Statistical differences in the TIMI frame counts were calculated by analysis of variance (ANOVA) with SPSS software. The chi-squared test was used for other statistical comparisons.

## Results

Thirty-eight patients were enrolled in the study. The baseline characteristics of the patients are summarised in Table 1. The mean time from CABG surgery was four years. Consistent with current literature, the incidence of LIMA graft side branches was 18% in our study. The mean age, gender and risk factors were similar between group 1 (LIMA without side branches) and group 2 (LIMA with side branches).

**Table 1:** Clinical Characteristics Of The Patients

	*Group 1: LIMA without side branch (n = 31)*	*Group 2: LIMA with side branch (n = 7)*	p*-value*
Mean age (years)	65.9 ± 7	66 ± 8	NS
Gender (male/female)	22/9	5/2	NS
Hypertension (*n*)	15	4	NS
Diabetes mellitus (*n*)	14	3	NS
Smoking (*n*)	20	5	NS

LIMA: left internal mammary artery; NS: not significant.

The mean TIMI frame count was 17.81 ± 5.3 for all patients. The mean LIMA graft TIMI frame count was 15.67 ± 2.3 in group 1 (*n* = 31) and 27.28 ± 3.4 in group 2 (*n* = 7). The difference between the two groups was statistically significant (*p* < 0.0001).

We observed that all side branches originated from the most proximal one-third of the LIMA’s length and descended to the anterior chest wall. The mean LIMA diameter of the proximal one-third segment was similar in the two groups. The diameter was 3.2 ± 0.2 mm in group 1 and 3.1 ± 0.2 mm in group 2 (*p* > 0.05). In group 2, the mean LIMA diameter of the proximal one-third segment (3.1 ± 0.2 mm) and mean diameter of the side branches (3.1 ± 0.2 mm) were similar (*p* > 0.05).

**Fig. 1.**
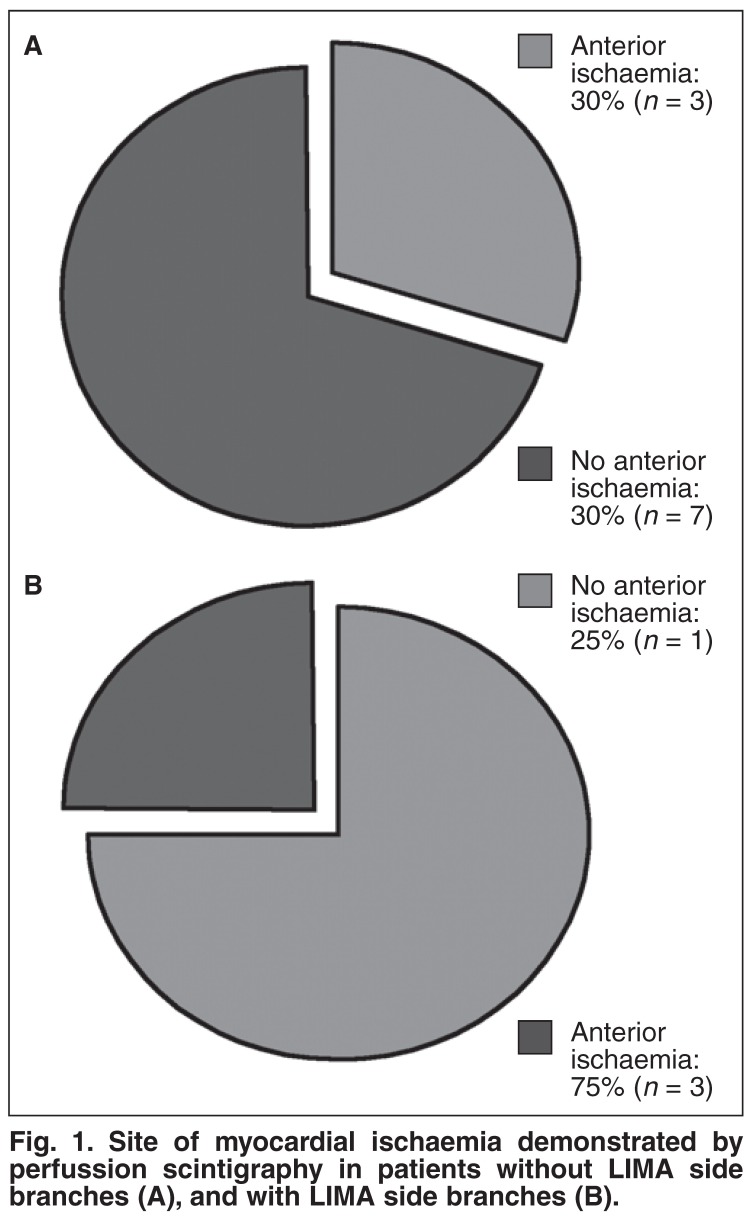
Site of myocardial ischaemia demonstrated by perfussion scintigraphy in patients without LIMA side branches (A), and with LIMA side branches (B).

Myocardial perfusion scintigraphy was performed in 14 patients (Table 2), 10 of whom were in group 1. Myocardial ischaemia of the anterior wall of the left ventricle was demonstrated in six patients, and in other sites of the left ventricle in the remaining patients. Three of the patients with anterior wall myocardial ischaemia were in group 1 (three of 10, 30%) and three were in group 2 (three of four, 75%) (Fig. 1).

**Table 2:** Indications Of Coronary Angiography In The Two Groups

	*Group 1: LIMA without side branch (*n* = 31)*	*Group 2: LIMA with side branch (*n* = 7)*
Treadmill test	12	2
MPS	10	4
ECG changes	9	1

LIMA: left internal mammary artery; MPS: myocardial perfussion scintigraphy.

## Discussion

The internal mammary arteries are the preferred choice as conduits for LAD revascularisation since they confer superior patency rates and improved survival compared to saphenous vein grafts.^11^ LIMA side branches to the chest wall (pectoralis branch) are relatively common.^1^,^2^ In a study by Singh and Sosa, postoperative angina may have been due to diversion of blood from the myocardium into the intercostal or pericardial branches of the internal mammary artery, which were not ligated at the time of surgery.^2^ Therefore, it is important to ligate all LIMA side branches during these operations.

We suggest that the TIMI frame count may be helpful in evaluating the effect of a side branch on the LIMA graft flow rate. In our study, we showed that the TIMI frame count in patients with LIMA grafts was significantly higher when LIMA side branches were present. This finding may indicate insufficient LIMA flow. Side branches, which cause an increase in the TIMI frame count in patients with LIMA grafts, may result in myocardial ischaemia. The TIMI frame count may be a useful way to identify insufficient coronary flow in patients with LIMA side branches. The measurement is simple, but there are no clear TIMI frame count cut-off values as yet to predict ischaemia. More studies using frame counts combined with other methods to demonstrate myocardial ischaemia are necessary.

From the available data, LIMA side-branch occlusion is unwarranted unless it is necessary to increase LIMA flow or reduce myocardial ischaemia.^10^ It is more likely that ischaemia is the result of compromised blood flow from another source, such as native coronary artery disease.^3^ There are several treatment methods for occluding side branches. The most popular method in the current literature is coil embolisation.^7^,^12^ Myocardial perfusion scintigraphy is often used to detect myocardial ischaemia. We believe that measurement of the TIMI frame count in patients with LIMA grafts before and after embolisation may demonstrate improvement in myocardial perfusion.

In our study, all side branches originated from the proximal one-third of the LIMA. We were not able to investigate the effect of distal side branches on the LIMA TIMI frame count. Since the diameters of the LIMA and side branches were similar in our study, we could not investigate how smaller side branches affect TIMI frame counts. Atherosclerosis of the distal left anterior descending artery may be a factor that decreases the TIMI frame count in LIMA patients, so flow rate differences may not solely be a result of side branches. Despite the small patient population in our study, the TIMI frame count was higher in patients with LIMA side branches and anterior wall myocardial ischaemia. Increases in TIMI frame count of a LIMA with a side branch should raise suspicion of myocardial ischaemia and further tests should be performed.

## Conclusion

Patent LIMA side branches are capable of compromising LIMA flow. However, side branches do not always cause myocardial ischaemia. The TIMI frame count is a simple tool to predict myocardial ischaemia in such patients. Large-scale studies are warranted to test the accuracy of this parameter.
